# MicroRNA Profiling of Self-Renewing Human Neural Stem Cells Reveals Novel Sets of Differentially Expressed microRNAs During Neural Differentiation *In Vitro*

**DOI:** 10.1007/s12015-023-10524-2

**Published:** 2023-03-14

**Authors:** Veronika Fedorova, Katerina Amruz Cerna, Jan Oppelt, Veronika Pospisilova, Tomas Barta, Marek Mraz, Dasa Bohaciakova

**Affiliations:** 1 Department of Histology and Embryology, Faculty of Medicine, Masaryk University, Brno, Czech Republic; 2 Department of Pathology and Laboratory Medicine, Division of Neuropathology, Perelman School of Medicine, University of Pennsylvania, Philadelphia, PA USA; 3 Central European Institute of Technology, Masaryk University, Brno, Czech Republic; 4 Department of Internal Medicine, Hematology and Oncology, University Hospital Brno and Faculty of Medicine, Masaryk University, Brno, Czech Republic; 5 International Clinical Research Center (ICRC), St. Anne’s University Hospital, Brno, Czech Republic

**Keywords:** Neural stem cells, microRNA, miRNA sequencing, Cell cycle, Human pluripotent stem cells

## Abstract

The involvement of microRNAs (miRNAs) in orchestrating self-renewal and differentiation of stem cells has been revealed in a number of recent studies. And while in human pluripotent stem cells, miRNAs have been directly linked to the core pluripotency network, including the cell cycle regulation and the maintenance of the self-renewing capacity, their role in the onset of differentiation in other contexts, such as determination of neural cell fate, remains poorly described. To bridge this gap, we used three model cell types to study miRNA expression patterns: human embryonic stem cells (hESCs), hESCs-derived self-renewing neural stem cells (NSCs), and differentiating NSCs. The comprehensive miRNA profiling presented here reveals novel sets of miRNAs differentially expressed during human neural cell fate determination *in vitro.* Furthermore, we report a miRNA expression profile of self-renewing human NSCs, which has been lacking to this date. Our data also indicates that miRNA clusters enriched in NSCs share the target-determining seed sequence with cell cycle regulatory miRNAs expressed in pluripotent hESCs. Lastly, our mechanistic experiments confirmed that cluster miR-17–92, one of the NSCs-enriched clusters, is directly transcriptionally regulated by transcription factor c-MYC.

**Graphical Abstract:**

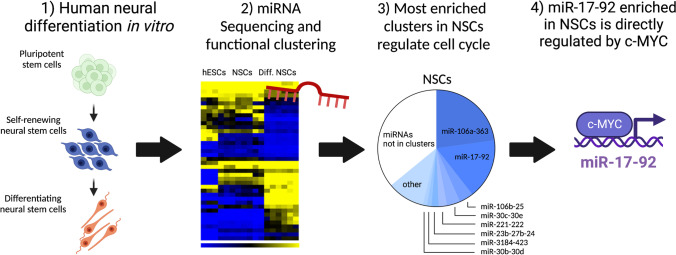

**Supplementary Information:**

The online version contains supplementary material available at 10.1007/s12015-023-10524-2.

## Highlights

- Upon differentiation from hESCs, self-renewing neural stem cells maintain fast proliferation and stem cell-like cell cycle properties
- MiRNA sequencing reveals novel sets of differentially expressed miRNAs during neural cell fate determination *in vitro*
- MiRNA clusters enriched in NSCs share the seed sequence with cell cycle regulatory miRNAs in pluripotent hESCs and one of them, miR-17–92, is directly transcriptionally regulated by c-MYC

**Supplementary Information:**

The online version contains supplementary material available at 10.1007/s12015-023-10524-2.

## Introduction

Rapid self-renewal and differentiation are two defining qualities of stem cells. In the last two decades, it has been repeatedly shown that the maintenance of stemness in human embryonic stem cells (hESCs) is directly linked to specific regulation of the cell cycle properties [1, 2]. Importantly, studies also show that with the onset of terminal differentiation, the G1 phase of the cell cycle is markedly prolonged, with underlying molecular mechanisms undergoing dramatic changes. And while a great amount of research has been done to understand the cell cycle biology of undifferentiated pluripotent stem cells, the maintenance of self-renewing capacity and the onset of differentiation in other multipotent stem cell types remains poorly described. Especially in the context of human brain development *in vivo*, where any deregulation of neural stem cell maintenance and function can lead to diseases such as microcephaly, epilepsy, or even neurodegeneration [3–5], it is essential to understand how they preserve their identity.

Increasing attention was recently brought to the role of microRNAs (miRNAs) in orchestrating self-renewal and differentiation of stem cells during development. MiRNAs are short, single-stranded, non-coding RNAs which regulate gene expression by silencing the translation of mRNAs. Mature miRNAs are incorporated into the RNA-induced silencing complex (RISC) to identify the target mRNA and mediate the repression of its translation and/or stability. Notably, a substantial fraction of miRNAs in the human genome is encoded in clusters that consist of two or more miRNA genes localized in the same genomic region with the same orientation and are generally transcribed as one unit [6]. Importantly, members of one miRNA cluster are often involved in molecular pathways leading to a similar biological outcome, thus having a cooperative effect on regulating the target genes [7]. Moreover, it has been shown that miRNAs from the same cluster often target functionally related genes. As there are around 2300 human miRNAs identified thus far, each affecting up to hundreds of target mRNAs, the influence of miRNA regulation on gene expression is extensive [8]. However, since the utilization of miRNAs depends on numerous biological parameters and settings, the transfer of knowledge about miRNA function between different cell types and conditions is limited.

Since their discovery, miRNAs and miRNA-mediated regulation of translation proved to be indispensable in cellular differentiation, including the development of the nervous system. Studies on mice show that the absence of miRNA processing enzymes Dicer1 or Argonaut2 leads to embryonic lethality [9, 10]. In the context of neurogenesis, the conditional deletion of Dicer1 has been found to impair the differentiation of neural stem cells (NSCs) [11–13]. Further supporting their importance, miRNAs were found to execute important functions in a precise timely manner to mediate cortical development. Specifically, miR-9, miR-137, miR-219, and let-7b were found to be involved in the regulatory loops of the TLX transcription factor, leading to the induction of differentiation of neural progenitors in mice [14–17]. MiR-9 and miR-124 were found to mediate neurodifferentiation through the inhibition of the RE-1 silencing transcript factor (REST), a major negative regulator of neurogenesis [18–20], and miR-125 and let-7 to be involved in a regulatory loop with LIN28, thus affecting the differentiation of NSCs [21]. Neuronal subtype specification, axon outgrowth, and dendritic arborization have also been found to be regulated by miRNAs [22–26]. Hence, a comprehensive study of the dynamics of miRNA expression during human neurodifferentiation is essential to understand the development and maintenance of neural cell types. Indeed, some of these molecular loops have also been described in human neural differentiation *in vitro* (comprehensively reviewed in [26, 27]). However, the miRNA profile of human NSCs remains, to this date, incomplete.

Here, we used a model of well-characterized human NSCs—multipotent, self-renewing stem cells of the nervous system derived from pluripotent human embryonic stem cells (hESCs) [28, 29]. We show that upon differentiation from hESCs, self-renewing NSCs maintain fast proliferation and stem cell-like cell cycle properties. Importantly, we further present comprehensive miRNA sequencing revealing novel sets of differentially expressed miRNAs during human neural cell fate determination *in vitro*. Notably, our data also indicate that miRNA clusters enriched in NSCs share the seed sequence with cell cycle regulatory miRNAs in pluripotent hESCs. Lastly, our mechanistic experiments confirmed that the cluster miR-17–92, one of the NSCs-enriched clusters, is directly transcriptionally regulated by transcription factor c-MYC also in self-renewing NSCs.

## Methods

### Cell Culture, Differentiation, and Sample Collection

Human ES cell line H9 (WA09) was passaged and maintained using standard feeder-free culture protocol on Matrigel-coated plates (Corning) in mTeSR^TM^1 (STEMCELL Technologies) and passaged using TrypLE (Thermofisher Scientific) as previously described in Raska et al., 2021 [30, 31]. For sample collection of self-renewing hESCs, cells were seeded at the density of 20,000/cm^2^ on cell culture plates (Day 0) coated with Matrigel and maintained in mTeSR^TM^1 medium (changed daily). Samples were harvested on Day 3.

Two NSC lines (CoMo-NSC) were derived from human embryonic stem cells (cell lines H9 and ESI-017) as described previously [28]. Self-renewing NSCs were cultured on the cell culture plates coated with poly-L-ornithine (Merck) and laminin (Thermo Fisher Scientific). Standard growth medium contained DMEM/F12, 1% Glutamax, 1% non-essential amino acids, 0.5% N2 supplement, 1% B27 supplement without vitamin A, 20 ng/mL FGF2 (fibroblast growth factor 2) recombinant human protein (Thermo Fisher Scientific), and 5 µL/mL of Zell shield cell culture contamination preventive solution (Minerva Biolabs). The medium was changed daily, and cells were passaged using Accutase (Thermo Fisher Scientific). For sample collection of self-renewing NSCs, cells were seeded at the density of 25,000/cm^2^ on cell culture plates (Day 0) coated with poly-L-ornithine and laminin and maintained in a standard growth medium with FGF2 (changed daily). Samples were harvested on Day 3. For terminal differentiation, NSCs were seeded at the density of 25,000/cm^2^ on cell culture plates (Day 0) coated with poly-L-ornithine and laminin. From Day 3, the cells were left to spontaneously differentiate in a standard growth medium without FGF2, and the medium was changed every second day. Cells were harvested on Day 14 of differentiation without FGF2.

### Growth Curve Construction and Cell Cycle Length Calculation

For the growth curve construction, cells were plated in equal amounts into four 96-well plates in at least 6 wells per plate. Every subsequent day, one of the 96-well plates was fixed with 4% paraformaldehyde, washed with PBS, and labeled with Hoechst 33,342 solution (Thermo Fisher Scientific) diluted to a final concentration of 5 µg/mL in PBS. Cell imaging was performed on an ImageXpress Micro XL automated epifluorescence microscope (Molecular Devices) using a Plan Fluor ELWD 20x/0.45 objective. The number of cell nuclei on acquired images was analyzed manually by ImageJ software (https://imagej.nih.gov/ij/). Cell cycle length was then calculated during 48 h between D2 and D4 using the formula: $Doubling \ time=\frac{48*\mathrm{ln}(2)}{\mathrm{ln}(\frac{number \ of \ cells \ on \ D4}{number \ of \ cells \ on \ D2})}$  

### Flow Cytometry and Cell Cycle Analysis

For the cell cycle analysis, cells were enzymatically harvested, washed with PBS, fixed using 1 mL of ice-cold 70% ethanol, and stored at 4 °C for at least 30 min. Before the flow cytometry analysis, fixed cells were washed twice with FACS buffer (0.5 M EDTA, 2% FBS, 1 × PBS), and the pellet was resuspended and incubated in 250 µL FACS buffer with 50 µL RNase A (0.1 mg/mL) for 30 min at 37 °C. Cell nuclei were then stained by propidium iodide (Thermo Fisher Scientific) at the final concentration of 50 µg/mL, incubated in the dark for 30 min at room temperature, and subjected to FACS analysis using BD FACS Canto II cytometer (Becton Dickinson). Data were collected for 10,000 events per sample. The analyses were performed with FlowJo 7.2.2 software (Tree Star).

### RNA Isolation and qPCR

Total RNA was isolated by RNA Blue reagent (Top-Bio) according to the manufacturer’s instructions based on the phenol–chloroform extraction principle [32]. For protein-coding gene expression analysis, the isolated RNA was transcribed to cDNA using Transcriptor First Strand cDNA Synthesis Kit (Roche) according to the manufacturer’s instructions. qPCR was performed from the cDNA samples using LightCycler 480 SYBR Green I Master kit (Roche) on LightCycler 480 II (Roche). For miRNA gene expression analysis, TaqMan MicroRNA Assays (Thermo Fisher Scientific) specific for selected mature miRNAs were used according to the manufacturer’s protocol. Ct values were calculated using the automated Second Derivative Maximum Method in LC480 software (Roche). The relative gene expression was calculated by normalization to glyceraldehyde 3-phosphate dehydrogenase (GAPDH) expression (for protein-coding genes) or to the average expression of two small nucleolar RNAs (RNU6B and RNU38B) for miRNAs as described previously [33]. Dots in the graphs represent individual biological experiments (*n* = 3 for hESCs; *n* = 6 for NSCs and *n* = 6 Diff.NSCs). Error bars represent SEM, *p < 0.05; **p < 0.01; ***p < 0.001; ****p < 0.0001. Primers and probes used in this work are listed in Table S1.

### Western Blotting

Western blot analysis was performed as described previously [34]. Briefly, cells were washed three times with PBS, lysed in lysis buffer (50 mM Tris–HCl [pH 6.8], 1% sodium dodecyl sulfate, 10% glycerol), and stored at − 70 °C until use. Equal amounts of total proteins were separated by SDS–polyacrylamide gel electrophoresis, transferred to a polyvinylidene fluoride membrane (Millipore), and proteins were immunodetected using the appropriate primary antibody followed by incubation with horseradish peroxidase-conjugated secondary antibody. Amersham ECL Prime western blotting Detection Reagent (GE Healthcare Life Sciences) was used to visualize antibody-antigen complexes. All used antibodies are listed in Table S2.

### Immunocytochemistry and Microscopy

Immunocytochemistry of NSCs and Diff.NSCs was performed as described previously [35]. Briefly, cells were fixed with 4% paraformaldehyde, permeabilized using 0.2% Triton X100 in 1 × PBS for 15 min and incubated with primary antibodies overnight at 4 °C. Secondary antibodies and Hoechst 33,342 were diluted in the permeabilization buffer and incubated with cells for 1 h at room temperature. After incubation, the slides were washed extensively with PBS, dried, and mounted onto microscopic slides with Mowiol 4–88 Reagent (Merck).

Samples were imaged with the inverted microscope Zeiss Axio Observer.Z1 with confocal unit LSM 800, equipped with solid state lasers (405, 488, 561 and 640 nm) and Plan-Neofluar 20x/0.50 AIR and Plan-Apochromat 63x/1.40 OIL objectives using ZEN Blue software (Zeiss). Images with 0.16 × 0.16 × 0.80 μm (20x) and 0.07 × 0.07 × 0.28 μm (63x) pixel size were acquired using GaAsP PMT detectors. The acquisition parameters for Alexa Fluor 405, 488, 568, and 647 were: 410–470, 497–553 nm, 565–617 nm, and 656–700 nm (emission wavelength range). Pixel dwell time was 1.03 μs (20x) and 1.47 μs (63x). The pinhole was set to 1 AU—1 μm (20x) and 0.6 μm (63x). Line average of 2 was applied to all channels.

### Chromatin Immunoprecipitation

Chromatin immunoprecipitation was performed according to Nelson et al., 2006 [36] with several modifications. Cells were cross-linked by adding formaldehyde to the cell culture medium to a final concentration of 1.42% and incubated for 15 min. Subsequently, formaldehyde was quenched using 125 mM glycine and cells were washed twice with PBS. Cells were then scraped and collected by centrifugation (200 g/3 min). Dry pellet was kept on -80 °C until analysis.

Pellet was lysed on ice in IP buffer (150 mM NaCl, 50 mM Tris–HCl (pH 7.5), 5 mM EDTA, 0.5% NP-40, 1% Triton-X-100) with protease inhibitors (5 μl/ml 0.1 M PMSF and 1 μl/ml 10 μgμl^−1^ leupeptin) and centrifuged (12,000 g/ 5 min/ 4 °C). Pellet was washed with IP buffer with protease inhibitors and resuspended in 1 ml of the same buffer. Following the lysis, chromatin was sonicated to obtain 400 – 600 bp long fragments, cleared by centrifugation (12,000 g/ 5 min/ 4 °C), and 80 μl of supernatant was used for DNA isolation using DNeasy Blood & Tissue kit (QIAGEN). Sonication efficiency was verified on the agarose gel. Subsequently, chromatin was divided into two fractions – negative control incubated with no antibody and ChIP sample incubated with 5 μl of c-MYC antibody (Cell Signaling – 5605) for 1 h on ice. Protein G Sepharose beads (Protein G Sepharose™ 4 Fast Flow, 17–0618-01, GE Healthcare) blocked with 10 μg/μl Salmon sperm DNA (UltraPure™ Salmon Sperm DNA Solution, Thermofisher Scientific) in IP buffer with protease inhibitors were added to chromatin samples and incubated overnight on 4 °C on a horizontal rotator.

The next day, protein G Sepharose beads were washed 5 times with IP buffer without protease inhibitors, and DNA was eluted with elution buffer (50 mM Tris pH 7.5, 1% SDS, 1 mM EDTA) 3 times by incubating 15 min/ 50 °C. Supernatants were pooled and incubated overnight on 65 °C to decross-link. DNA was isolated from the decross-linked samples using DNeasy Blood & Tissue kit (QIAGEN). PCR was performed using Taq PCR reagents (TopBio) according to manufacturer’s instructions with primers flanking E-box regions in the promoter region of miR-17–92 cluster and Apex1 as a positive control to c-MYC binding. Negative control represents genomic region Homo sapiens ribosomal protein L32 pseudogene with no predicted binding site for c-MYC. PCR products were visualized on 2% agarose gel.

### Small RNA Library Preparation, Sequencing, and Data Processing

For miRNA sequencing, a set of hESCs, NSCs, and Diff.NSCs was cultivated in duplicates, and RNAs were pooled to get the final sets used for library preparation (hESC *n* = 3; NSCs *n* = 8; Diff.NSCs = 6). Total RNA was isolated with RNA Blue, RNA quality was assessed by TapeStation 2200 (#5067–5576 RNA Screen Tape; Agilent Technologies), and only samples with RINe values ≥ 9 were used for library preparation. NEBNext Multiplex Small RNA Library Prep Set for Illumina (#E7300S, #E7580S Set1, 2; New England Biolabs) was used to prepare libraries for further sequencing according to the manufacturer’s instructions. Briefly, 800 ng of total RNA was used to create size-selected small RNA libraries (size selection with 6% PAGE gel). The sequencing was performed with 2.0 pM library using the NextSeq500/550 High Output Kit v2.5 (#20,024,906; 75 cycles; Illumina).

The quality of the raw sequencing data was assessed using FastQC (v0.11.9) (https://www.bioinformatics.babraham.ac.uk/projects/fastqc/). Minion and Swan (Kraken package, v16.098) [37] were used to scan and identify adaptor sequences which were subsequently removed by Cutadapt (v2.5) [38]. Only adapter-containing reads were kept for further processing. The adapter-trimmed reads were further processed using the following steps: 1) Removal of very low-quality read ends (Phred < 5), 2) Keeping only reads with a Phred score of 10 over at least 85% of the length, 3) Only reads within 16–27 bp were kept as potential miRNA reads. FASTX-Toolkit (v0.0.14) (http://hannonlab.cshl.edu/fastx_toolkit/) was used for the quality filtering; the rest of the steps were performed by Cutadapt (v2.5) [38] and bash scripting. The quality of the final pre-processed reads was assessed by overall mapping rates to the human reference genome (hg38) [39], and the general quality of the pre-processed reads was assessed by Bowtie (v1.3.1) [40]. Reads mapping to rRNAs, tRNAs, snoRNAs, snRNAs, or YRNAs [39] with fewer mismatches than to miRNAs (miRBase; v22.1) [41] were excluded. The raw miRNA expression levels were quantified by seqcluster (v1.2.8) [42] and seqbuster/miraligner (v3.5) [43]. R (v3.6.3) (https://www.r-project.org/) was used for further evaluation and visualization of the data. Differential expression was calculated with DESeq2 (v1.24.0) [44] and edgeR (v3.26.8) [45]. A total number of 998 miRNAs was identified. For further analysis, we selected miRNAs with normalized counts higher than 50 in at least two samples of one cell type, resulting in the detection of 347 miRNAs.

### Quantification and Statistical Analysis

Data analyses were performed using GraphPad Prism version 8 for Windows, GraphPad Software, San Diego, California USA, www.graphpad.com. Two-tailed unpaired parametric Student’s t-test was performed, and differences were considered statistically significant at *p < 0.05; **p < 0.01, ***p < 0.001 and ****p < 0.0001. All data are presented as mean and ± SEM and plotted as a bar graph with depicted individual values as dots.

## Results

### Self-Renewing NSCs Maintain Fast Proliferation and Stem Cell-Like Cell Cycle Properties

**Fig. 1:**
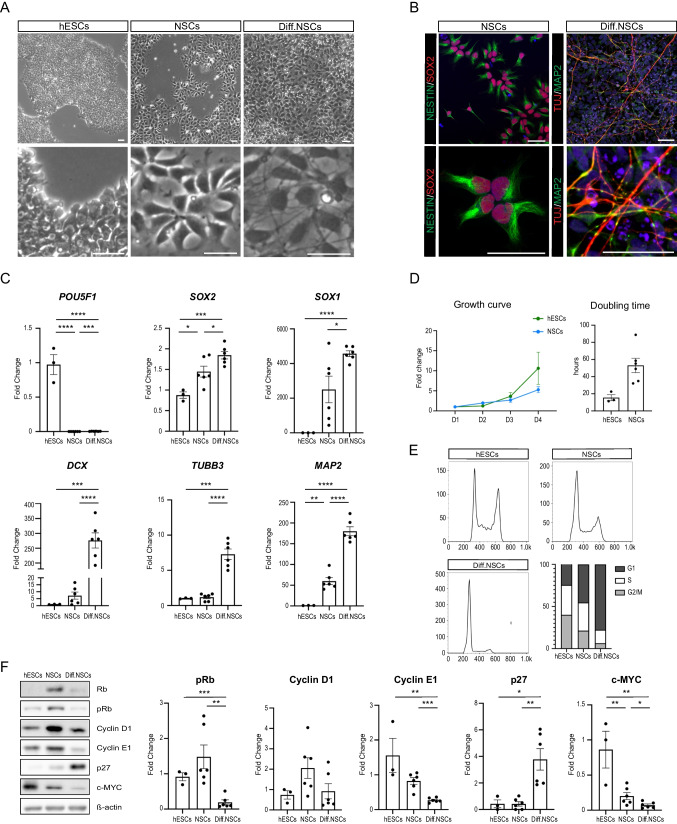
Upon differentiation from hESCs, self-renewing NSCs maintain fast proliferation and stem cell-like cell cycle properties. **(A)** Representative brightfield microscopy images showing the morphology of hESCs, NSCs, and Diff.NSCs. Scale bar = 50 μm. **(B)** Representative immunocytochemistry images of the markers of NSCs (SOX2 and NESTIN) in NSCs (left), and neuronal markers (TUJ and MAP2) in Diff.NSCs (right). Scale bar = 50 μm. **(C)** qPCR analysis of markers of hESCs (*POU5F1, SOX2*), NSCs (*SOX2, SOX1*), and neural markers (*DCX, TUBB3, and MAP2*) in hESCs, NSCs, and Diff.NSCs. **(D)** Growth curve analysis of hESCs and NSCs between Day 1 – Day 4 (left) and average doubling time of hESCs and NSCs calculated based on the growth curve analysis (right). **(E)** Cell cycle profile of hESCs, NSCs and Diff.NSCs and the quantification of cells in the phases of the cell cycle of each cell type (bottom right). **(F)** Western blot analysis of the cell cycle regulators Rb, pRb (Thr 821, Thr 826), Cyclin D1, Cyclin E1, p27, and c-MYC in hESCs, NSCs, and Diff.NSCs

To initiate our studies of self-renewing properties of human NSCs, we first aimed to characterize our model cell types. For this purpose, we used two independent cell lines of self-renewing CoMo-NSCs derived from two hESC lines (H9 and ESI-017; here referred to as “NSCs”; [28]) and their differentiating, non-self-renewing, counterparts (here referred to as “Diff.NSCs”). We also included undifferentiated hESCs (H9 cell line) in all our analyses as a reference pluripotent stem cell line. As shown in Fig. 1A, all experimental cell types displayed the typical morphology described previously [28, 46]. Immunocytochemistry confirmed that undifferentiated NSCs expressed characteristic markers such as SOX2 and NESTIN, whereas, upon induction of differentiation, Diff.NSCs dramatically changed their morphology and began to express neuronal markers TUJ and MAP2 (Fig. 1B). We further determined the expression of other pluripotency (*POU5F1, NANOG*) and differentiation-associated (*SOX2, SOX1, DCX, TUBB3, and MAP2*) genes using qPCR (Fig. 1C and Fig. S1A). Data show that the expression of pluripotency-associated genes *POU5F1 and NANOG* significantly decreased with the differentiation of hESCs into NSCs. On the contrary, the expression of NSC-related markers *SOX2* and *SOX1* significantly increased with the onset of differentiation. The expression of both immature and mature neuronal markers (*DCX, TUBB3, and MAP2, respectively*) increased and showed significantly high levels in Diff.NSCs. This initial analysis confirmed a successful onset of neural differentiation *in vitro*.

We then proceeded to the analysis of cell cycle parameters of the model cell types since the fast proliferation, short G1 phase, and specific expression of cell cycle regulators were previously directly linked to the maintenance of stemness [1, 47–49]. We first analyzed the growth rate of hESCs, and NSCs and calculated the cell cycle length of each cell type. Data show a gradual prolongation of growth rate with ongoing differentiation. Specifically, we show that self-renewing hESCs retain the fastest growth rate with the average cell cycle length of 15–16 h (Fig. 1D), confirming the previous observations [1]. We further reveal that self-renewing NSCs prolong the cell cycle length, yet they maintain relatively fast proliferation (average cell cycle duration of 53 h). The cell cycle length is further slowed down upon initiation of terminal differentiation. Evaluation of the cell cycle profile using flow cytometry and quantification of the percentage of cells in each phase of the cell cycle (Fig. 1E and Fig. S1B) support this observation and show that cell cycle properties in NSCs acquire an intermediate phenotype between pluripotency and commitment to terminal differentiation. Specifically, we found that in hESCs, the G1 phase is very short, with only 25.87% (± 2%) of cells residing in this phase. With the onset of differentiation, the number of cells in G1 phase gradually increased to 42.45% (± 1.76%) in self-renewing NSCs and 65.98% (± 2.19%) in Diff.NSCs. On the contrary, the number of cells in S phase decreased from 37.67% (± 0.59%) in hESCs to 31.33% in NSCs (± 1%) and 13.52% (± 1.11%) in Diff.NSCs. Finally, the proportion of cells in G2/M phase was reduced from 42.77% (± 3.82%) in hESCs, to 20.08% (± 1.23%) in NSCs and further to 5.46% (± 0.71%) in Diff.NSCs. Lastly, the western blot analysis of selected G1/S phase transition regulators (cyclin D1, cyclin E1, p27, c-MYC, and pRb protein) in self-renewing NSCs (Fig. 1F) showed that their expression is mostly comparable to hESCs and only changes in differentiating NSCs. Specifically, we found that pRb phosphorylation and the expression of cyclin D1 were highest in self-renewing NSCs suggesting a fast transition from G1 to the S phase in NSCs. This is further supported by the levels of cyclin E1 and c-MYC, which remained high in comparison to Diff.NSCs and by the level of CDK inhibitor p27, which remained low in self-renewing NSCs and only significantly increased upon induction of terminal differentiation. Thus, our results confirm previous studies, which show that undifferentiated hESCs have a unique cell cycle regulatory mechanism characteristic for its fast proliferation and short G1 phase. Importantly, data further show that self-renewing NSCs, while already committed to differentiation towards neural cell fate, also retain some of the cell cycle properties typical for stem cells. These properties are then undetectable upon induction of terminal differentiation, suggesting that also in hESC-derived NSCs, specific cell cycle properties are likely connected to the maintenance of their phenotype.

### miRNA Sequencing Reveals Novel Sets of Differentially Expressed miRNAs during Neural Cell Fate Determination *In Vitro*

**Fig. 2:**
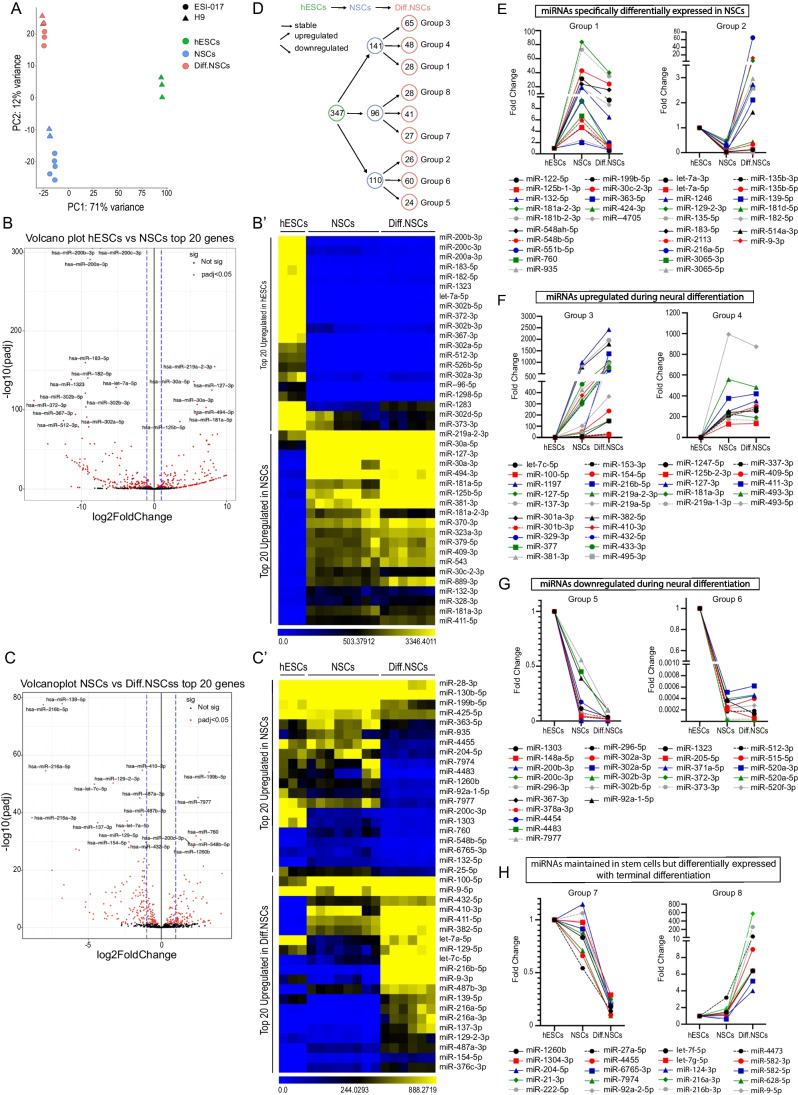
miRNA sequencing reveals novel sets of differentially expressed miRNAs during neural cell fate determination *in vitro*. **(A)** Principal component analysis (PCA) showing the variability between hESCs, NSCs, and Diff.NSCs of two cell lines (H9, ESI-017). **(B)** Volcano plot showing differential miRNA expression. Twenty most significantly upregulated and downregulated differentially expressed miRNAs in NSCs vs. hESCs are named. Red dots – differentially expressed miRNA (Adjusted p-value < 0.5, |Fold change|> 0). **(B’)** Heatmap showing 20 most upregulated miRNAs in hESCs and 20 most upregulated miRNAs in NSCs in hESCs, NSCs and Diff.NSCs samples. **(C)** Volcano plot showing differential miRNA expression. 20 most significantly upregulated and downregulated differentially expressed miRNAs in NSCs vs. Diff.NSCs are named. Red dots – differentially expressed miRNA (Adjusted p-value < 0.5, |Fold change|> 0). **(C’)** Heatmap showing 20 most upregulated miRNAs in NSCs and 20 most upregulated miRNAs in Diff.NSCs in hESCs, NSCs, and Diff.NSCs samples. **(D)** Scheme showing the categorization of miRNAs into groups based on their expression pattern during neural differentiation and the number of miRNAs in each group. **(E)** The expression of the top 15 miRNAs from Group 1 in hESCs, NSCs, and Diff.NSCs (left) and the expression of top 17 miRNAs from Group 2 in hESCs, NSCs, and Diff.NSCs (right). **(F)** The expression of top 20 miRNAs from Group 3 in hESCs, NSCs, and Diff.NSCs (left) and the expression of top 10 miRNAs from Group 4 in hESCs, NSCs, and Diff.NSCs **(G)** The expression of top 16 miRNAs from Group 5 in hESCs, NSCs, and Diff.NSCs (left) and the expression of top 10 miRNAs from Group 6 in hESCs, NSCs, and Diff.NSCs (right) **(H)** The expression of top 10 miRNAs from Group 7 in hESCs, NSCs, and Diff.NSCs (left) and top 10 miRNAs from Group 8 in hESCs, NSCs, and Diff.NSCs (right)

With the cell cycle properties of our model cell types characterized, we proceeded to the analysis of miRNAs involved in the neural differentiation *in vitro*. Curiously, despite miRNAs being crucial for normal stem cell self-renewal and cellular differentiation, their composition in self-renewing NSCs, and how their expression changes with the onset of terminal differentiation remained, to a large extent, undescribed. Thus, to identify miRNAs associated with the identity of NSCs, we performed miRNA profiling (Illumina) in all our model cell types and verified the expression of selected miRNAs using qPCR. As shown in Fig. 2A, Principal component analysis (PCA) indicated that each cell type clustered together in a different localization across the PCA plot indicating significant and consistent variability between analyzed samples. Subsequently, differential gene expression analysis allowed us to identify the most significantly upregulated (Adjusted p-value < 0.05, Fold Change > 1) and downregulated (Adjusted p-value < 0.05, Fold Change < 1) miRNAs in each analyzed cell type. As visualized in the volcano plot (Fig. 2B) and heatmap (Fig. 2B’), the most significantly upregulated miRNAs in pluripotent hESCs were, among others, miR-302-367 cluster (Fig. S2A), and miR-200 family members previously identified by us and others [50–54]. In comparison to pluripotent hESCs, most significantly upregulated miRNAs in self-renewing NSCs included some of the miRNAs previously associated with the proliferation of NSCs (miR-181, miR-30) [55, 56]. Importantly, our statistical analysis also identified numerous other miRNAs that have, thus far, not been linked to the neural differentiation of pluripotent stem cells. Substantial differences were also observed when self-renewing NSCs were compared to Diff.NSCs (Fig. 2C and C’), with well-known miRNAs miR-124, miR-9 and miR-219-a-2 being significantly upregulated only in terminally differentiating NSCs (Fig. S2A). Notably, besides these 3 miRNAs, the list of differentially expressed miRNAs included less well-characterized ones that were, to a large extent, not previously associated with terminal differentiation. A complete list of differentially expressed miRNAs can be found in Supplementary Table S3.

Next, we grouped significantly enriched miRNAs based on the trend in their expression during neural differentiation *in vitro.* Each miRNA was categorized based on a set of parameters (Adjusted p-value and log2FoldChange) for differences in miRNA expression between i) hESCs vs. NSCs (here referred to as p-adj1 and log2FoldChange1), and ii) NSCs vs. Diff.NSCs (here referred to as p-adj2 and log2FoldChange2). Significantly upregulated (Adjusted p-value < 0.05, log2FoldChange >  + 0.6) and downregulated (Adjusted p-value < 0.05, log2FoldChange > -0.6) miRNAs were identified, leaving remaining miRNAs considered as stable. As schematized in Fig. 2D, with this approach, we were able to identify which miRNAs were i) differentially expressed specifically in self-renewing NSCs (Group 1 and Group 2) (Fig. 2E), ii) differentially expressed during neural differentiation (Group 3, Group 4, Group 5 and Group 6) (Fig. 2F-G); and iii) maintained in stem cells but differentially expressed with the onset of terminal differentiation (Group 7 and Group 8) (Fig. 2H). Criteria for Adjusted p-value and log2FoldChange for each group can be found in Supplementary table S4 and a complete list of miRNAs in each category in Fig. S2B-E. This categorization of miRNAs further confirmed that besides a handful of well-studied miRNAs, the majority of miRNAs (and their relevant targets) remain unknown in the context of human neural differentiation and could be explored in future studies.

### miRNA Clusters Enriched in NSCs Share the Seed Sequence with Cell Cycle Regulatory miRNAs in Pluripotent hESCs

**Fig. 3:**
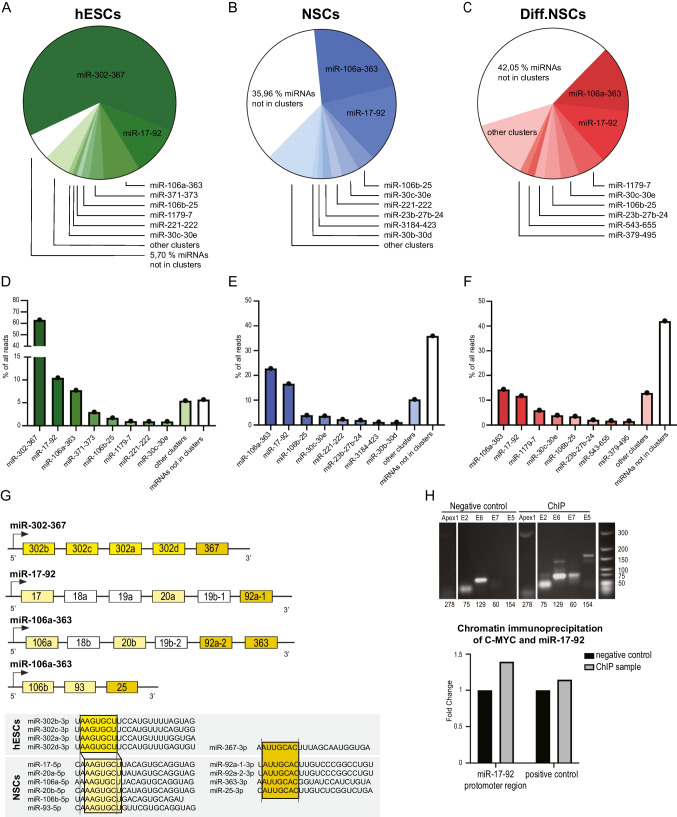
miRNA clusters enriched in NSCs share the seed sequence with cell cycle regulatory miRNAs in pluripotent hESCs and are directly transcriptionally regulated by c-MYC. **(A)(D)** Representation of miRNA clusters in hESCs. **(B)(E)** Representation of miRNA clusters in NSCs **(C)(F)** Representation of miRNA clusters in Diff.NSCs. **(G)** Scheme showing the similarities among the seed sequences of miRNAs from clusters enriched in hESCs and NSCs. **(H)** PCR analysis of four E-box regions in the promoter region of miR-17–92 cluster after chromatin immunoprecipitation (top). Quantification of chromatin immunoprecipitation analysis of c-MYC and miR-17–92 promoter region in negative control and ChIP sample compared to the positive control of c-MYC binding

Thus far, miRNA profiling data allowed us to identify i) individual miRNAs specifically enriched in all three analyzed cell types as well as ii) groups of miRNAs that show specific expression patterns during human neural differentiation *in vitro*. Lastly, to complement these analyses of individual miRNAs, we assessed the expression pattern of miRNAs that are transcribed together in clusters. Indeed, it has been previously shown that the pluripotent stem cell cycle is largely regulated by specific clusters of miRNAs with specific target-determining seed sequences defined as nucleotides in positions 2–8 of the mature miRNA [57]. We thus hypothesized that such analysis could reveal if any of the miRNA clusters are also specifically enriched in self-renewing or differentiating NSCs. We thus quantified the number of normalized reads of all miRNAs belonging to annotated clusters and subsequently calculated the relative contributions of these miRNAs to the complete population of miRNAs in hESCs, NSCs, and Diff.NSCs. As shown in Fig. 3A and D, the majority of miRNAs in hESCs (about 63%) belong to the miR-302–367 cluster, confirming previously published data on cell cycle regulatory miRNAs in mouse ES cells [57]. Importantly, our data now show that with differentiation to self-renewing NSCs, the expression of miR-302–367 cluster significantly decreases while the expression of the other three clusters becomes the most prominent in this cell type (Fig. S3A). Namely, the expression of the miR-106a-363 cluster becomes the most abundantly expressed cluster in NSCs (about 23%), followed by the miR-17–92 cluster comprising 17% of miRNAs and the miR-106b-25 cluster comprising 4% of miRNAs. Together these clusters group over 40% of all miRNA molecules expressed in NSCs (Fig. 3B and E). With the onset of terminal differentiation, these clusters become less enriched, and other miRNA clusters such as miR-1179–7, miR-543–655, and miR-379–495 become prominently expressed in differentiating NSCs (Fig. 3C and F). In general, we observed that miRNAs in Diff.NSCs are distributed across more clusters, and there is no longer any cluster as prevalent as clusters in analyzed stem cells. A complete list of miRNAs in each cluster can be found in Fig. S3B.

Additionally, to analyze the representation of miRNA families, we also assigned miRNAs to miRNA families defined based on the seed sequence (Fig. S3C). This shows that the vast majority (62,8%) of miRNAs in hESCs belong to the miR-302 family, 9% belong to the miR-17 family, and 5.3% to the miR-25 family. In NSCs, 40% of miRNAs are members of 3 miRNA families – miR-25 (14%), miR-30 (13%) and miR-17 (13%). With the initiation of terminal differentiation, the composition changes, with 13% of miRNAs being in the miR-17 family, 13% in the miR-9 family, and 10% in the miR-30 family. Similar to what we observed in the case of clusters, in Diff.NSCs miRNAs seem to be distributed across more miRNA families than in hESCs and NSCs.

Interestingly, upon investigating the fundamentals of the most significant clusters from each cell type, we noticed that clusters specifically enriched in hESCs and NSCs have similarities in their seed sequences. As schematized in Fig. 3G, the seed sequence of miR-367 (enriched in hESCs) is identical to the seed sequence of miR-92 and miR-363 (enriched in NSCs). Moreover, the seed sequence of miR-302 (enriched in hESCs) is highly similar to that of miR-17 and miR-106a (enriched in NSCs). This observation suggests that only a small number of seed sequences shared across clusters in hESCs and NSCs seem to regulate the stem cell-specific properties in different stem cell types. Additionally, since it is the seed sequence that determines the target mRNA, we speculate that these clusters might be involved in regulating the same processes, including the precise regulation of self-renewal. This, however, remains to be explored in future studies.

### miR17-92 Cluster Enriched in NSCs is Directly Transcriptionally Regulated by c-MYC

Lastly, to mechanistically link one of the most abundantly expressed clusters in self-renewing NSCs (miR-17–92) with its putative transcriptional factor, we searched the literature and found that c-MYC directly transcriptionally regulates miR17-92 cluster in other cell types such as lymphoma cells, leukemia cells, fibroblasts or cytotrophoblasts [58–60]. To analyze this regulation in self-renewing NSCs, we performed chromatin immunoprecipitation on endogenously expressed miR-17–92. As shown in Fig. 3H, transcription factor c-MYC directly binds to the promoter region of miR-17–92 and regulates its expression. This data prove that the fast proliferation, which is part of the human neural stem cell phenotype, is also maintained by c-MYC via miR-17–92 cluster expression.

## Discussion

Here, we report the miRNA expression profile of self-renewing hESCs-derived NSCs and its connection to the cell cycle regulation and the maintenance of their self-renewing phenotype. We characterize how cell cycle properties of pluripotent stem cells gradually change with differentiation and demonstrate that self-renewing NSCs maintain an intermediary phenotype retaining some of the cell cycle properties typical for stem cells. Importantly, we also performed extensive miRNA profiling of all analyzed cell types and described novel sets of differentially expressed miRNAs during neural differentiation *in vitro*. Lastly, we show that miRNA clusters enriched in human self-renewing NSCs share the seed sequence with cell cycle regulatory miRNAs in pluripotent stem cells and one of these, miR-17–92, is directly transcriptionally regulated by c-MYC.

Self-renewal and differentiation are two defining attributes of stem cells. So far, these features have been extensively studied in pluripotent stem cells, where it has been shown that a specific cell cycle regulation is critical for hESCs to maintain fast proliferation and thus remain undifferentiated (reviewed in [61, 62]). This fast proliferation has been mainly attributed to the short G1 phase and specific expression of cell cycle regulators, such as hyperphosphorylated pRb protein, and low levels of D-type cyclins and CDK inhibitors [63–65]. It has also been shown that upon induction of terminal differentiation, the cell cycle is markedly prolonged with the prominent extension of the G1 phase [66–68]. Importantly, several studies proved that by modulation of cell cycle length or levels of cell cycle regulators, differentiation could be postponed or induced [47–49, 69–71]. Similarly to the situation in hESCs, studies have shown that the length of the G1 phase also influences the differentiation of neural progenitors, where lengthening of the G1 phase leads to their differentiation [72]. Moreover, the overexpression of p27 or the ablation of cyclin D1 was found to promote neurogenesis in developing mouse cortex [73, 74]. Here, we confirm previously reported data and show that undifferentiated hESCs have a unique cell cycle regulatory mechanism characteristic for its fast proliferation and short G1 phase [1]. Importantly, data further show that our self-renewing NSC lines, while already committed towards neural cell fate, also retain some of the cell cycle properties typical for stem cells, such as short G1 phase, large S phase, and relatively short G2 phase. These properties are then undetectable upon induction of terminal differentiation, with the majority of cells found in the G1 phase [61]. Our data thus suggest that also in hESC-derived NSCs, specific cell cycle properties are likely connected to the maintenance of their self-renewing phenotype.

Despite the importance of cell cycle regulation and the apparent contribution of miRNAs to this process, extensive miRNA profiles during human neural differentiation remain scarce. In mouse models, miRNA profiling has been performed on the developing mouse cortex, where the comprehensive miRNA expression was determined in NSCs, differentiating progenitors and newborn neurons [75]. A recent study on a different model reveals the dynamics of miRNA expression during Drosophila neurogenesis [76]. Furthermore, miRNA profiling of mouse ESCs (mESCs)-derived neural progenitor cells reported by Marson et al. provided a comprehensive list of miRNAs and miRNA clusters enriched in this cell type [57]. Their study shows that mESCs-derived neural progenitors have upregulated let-7, miR-9, and miR-124, suggesting that, unlike our NSCs, their cell type already has limited self-renewing capacity. Curiously, no such study has been, to this date, performed on human pluripotent stem cells-derived self-renewing NSCs. In a study by Stappert et al., miRNAs in neural differentiation of hESCs were verified by qPCR and several new miRNAs, such as miR-153, miR-324, and miR-181a, were reported in NSCs [25]. MiRNA array expression analysis was also done by Liu et al., 2019 [62]. This study has not specifically addressed the role of miRNA in human self-renewing NSCs, but the results indicate that miR-7 plays an essential role in neurogenesis. Another study by Kulcenty et al. has also used the miRNA array approach to identify the upregulation of miR-10, miR-30, and miR-9 families in human pluripotent stem cells derived NSCs [77]. Our results now significantly extend these studies by showing a comprehensive list of miRNAs that are differentially expressed during human neural cell fate determination *in vitro*. First, we confirm previous results in hESCs, where the miR-302–367 cluster is considered the master regulator of hESCs self-renewal. We then extend the list of miRNAs specifically expressed in human NSCs and show that except for miR-181 and miR-30, there are numerous other miRNAs with unknown functions during neural specification. Finally, for the first time, we report miRNA expression patterns from pluripotent hESCs to differentiating NSCs. We divided these miRNAs into eight groups which can now serve as a comprehensive platform for future studies. It is of note that miRNA profiling is currently limited to three isolated samples – one from each differentiation stage. Collection and analysis of more differentiation time points could reveal unique dynamics of miRNA expression during human neural differentiation *in vitro*.

Lastly, in addition to individual miRNAs and groups of miRNAs, we also analyzed miRNA clusters since they are often regulated by the same transcription factors and target similar mRNAs. To our best knowledge, the only study focused on miRNA cluster enrichment analysis was performed by Marson et al. (2008) [57]. This study brought novel insights into mESCs biology and directly linked miRNAs to the core transcriptional network of mESCs [57]. Several other studies of mainly animal models then focused specifically on individual clusters: the role of the miR-17–92 cluster was studied in the developing rat and mouse cortex, where it has been shown that it controls the development of neural stem cells as well as the axonal outgrowth by targeting PTEN [78–80]. MiRNA cluster miR-106b-25 was found to be involved in the regulation of NSCs proliferation in mouse primary cultures [81]. Interestingly, in a recent study, Favaloro et al. demonstrated that the miR-17–92 cluster, together with miR-106a-363 and miR-106b-25 clusters, are enriched in NSCs isolated from mice brains [82]. Functional experiments further showed that mouse NSCs with miR-17–92 deletion showed reduced proliferation *in vitro* [82]. However, characterization of the expression of miRNA clusters during human neural differentiation is, to date, lacking. Our study now complements previous findings by introducing miRNA clusters specifically enriched in human NSCs. Moreover, we show that they share the seed sequence with cell cycle regulatory miRNAs in pluripotent hESCs**.** Since the seed sequence is one of the parameters that determine the target mRNA, we speculate that these clusters might be involved in the regulation of the same process, including the tight regulation of the cell cycle. Functional experiments demonstrating the fate of NSCs upon deletion or overexpression of miR-17–92 remain to be performed. Additionally, we noticed that the number of miRNAs that belong to the same cluster decreases with the extent of differentiation, leading to a larger number of clusters enriched in differentiating NSCs. One can speculate that this may be one of the regulatory features specifically found in stem cells. This, however, remains to be supported experimentally using more stem cell types in future studies.

Lastly, our mechanistic experiment shows that one of these clusters, miR-17–92, is directly transcriptionally regulated by c-MYC. This has been previously reported for lymphoma and leukemia cell lines by Li et al., 2014 [58]. Here we show that this regulation is also functional in human self-renewing NSCs. And while pathological activation of c-MYC is associated with tumorigenesis [83–85], non-transformed c-MYC-expressing cells, including NSCs, need to implement its tight multilevel regulation [83, 86]. Importantly, our previous study demonstrated that both used NSC lines (H9 and ESI-017 Co-Mo NSCs) show favorable safety profiles and do not form tumors after *in vivo* grafting into over 40 rat and 3 pig animal models [28]. This data suggest that, similarly to human development *in vivo*, the tight regulation of c-MYC is naturally established during the *in vitro* differentiation of karyotypically normal human pluripotent stem cells.

Altogether, our data provide a systematic and comprehensive characterization of miRNA representation in hESCs, NSCs, and differentiating NSCs. Our findings support previously published reports about several already described miRNAs and introduce new ones that have not yet been studied in the context of human neural differentiation and the phenotype of NSCs. Furthermore, we show that upon differentiation of hESCs, NSCs enriched cluster miR-17–92 is directly regulated by c-MYC and shares seed sequence similarities with hESCs-specific miRNA cluster miR-302–367. Moreover, our data point to an interesting observation that has not yet been examined in the field of miRNA or stem cells: both in pluripotent and multipotent stem cells, miRNAs are preferentially localized and expressed in clusters. Since the importance of miRNA clustering in cells is still not fully understood, the biological relevance of this phenomenon remains to be elucidated [7].

## Conclusion

MiRNA profiling of self-renewing neural stem cells derived from human pluripotent stem cells reveals novel sets of differentially expressed miRNAs during neural differentiation *in vitro*. We also show that miRNA clusters enriched in self-renewing neural stem cells share the seed sequence with cell cycle regulatory miRNAs in pluripotent stem cells and are directly transcriptionally regulated by c-MYC.

## Supplementary Information

Below is the link to the electronic supplementary material.

Supplementary file1 Supplementary Figure 1: Upon differentiation from hESCs, self-renewing NSCs maintain fast proliferation and stem cell-like cell cycle properties. **(A)** qPCR analysis of pluripotency marker *NANOG* in hESCs, NSCs, and Diff.NSCs. **(B)** Cell cycle profile of hESCs, NSCs, and Diff.NSCs. (PDF 433 kb)

Supplementary file2 Supplementary Figure 2: miRNA sequencing reveals novel sets of differentially expressed miRNAs during neural cell fate determination *in vitro*. **(A)** qPCR analysis of the expression of pluripotency and differentiation-related miRNAs in hESCs, NSCs, and Diff.NSCs. **(B)** Complete list of miRNAs specifically differentially expressed in NSCs (Group 1 and Group 2). **(C)** Complete list of miRNAs upregulated during neural differentiation (Group 3 and Group 4). **(D)** Complete list of miRNAs downregulated during neuronal differentiation (Group 5 and Group 6). **(E)** Complete list of miRNAs maintained in stem cells but differentially expressed with terminal differentiation (Group 7 and Group 8). (PDF 542 kb)

Supplementary file3 Supplementary Figure 3: miRNA clusters enriched in NSCs share the seed sequence with cell cycle regulatory miRNAs in pluripotent hESCs and are directly transcriptionally regulated by c-MYC **(A)** Expression of selected clusters enriched in each cell type throughout the neural differentiation in hESCs, NSCs, and Diff.NSCs. **(B)** Table of miRNAs included in each cluster. **(C)** Representation of miRNA families in hESCs, NSCs, and Diff.NSCs (PDF 1743 kb)

Supplementary file4 (DOCX 6956 kb)

Supplementary file5 (XLSX 74 kb)

## Data Availability

Processed data are available upon reasonable request. All sequencing data are available in the SRA database under the BioProject number: PRJNA942316, ID: 942316 (https://www.ncbi.nlm.nih.gov/sra/PRJNA942316).
