# Supplementary figures and images for: MicroRNA Profiling of Self-Renewing Human Neural Stem Cells Reveals Novel Sets of Differentially Expressed microRNAs During Neural Differentiation In Vitro
Source: Stem Cell Rev Rep. 2023 Mar 14;19(5):1524–39. doi: 10.1007/s12015-023-10524-2 (PMC10366325; doi:10.1007/s12015-023-10524-2)

A

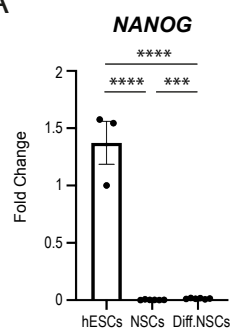

B

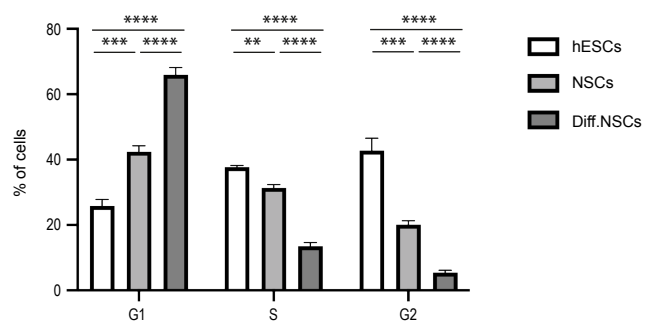

Supplement: Supplementary file 1 — Supplementary file1 Supplementary Figure 1: Upon differentiation from hESCs, self-renewing NSCs maintain fast proliferation and stem cell-like cell cycle properties. (A) qPCR analysis of pluripotency marker NANOG in hESCs, NSCs, and Diff.NSCs. (B) Cell cycle profile of hESCs, NSCs, and Diff.NSCs. (PDF 433 kb) [file 12015_2023_10524_MOESM1_ESM.pdf]
