# Supplementary material for: MicroRNA Profiling of Self-Renewing Human Neural Stem Cells Reveals Novel Sets of Differentially Expressed microRNAs During Neural Differentiation In Vitro
Source: Stem Cell Rev Rep. 2023 Mar 14;19(5):1524–39. doi: 10.1007/s12015-023-10524-2 (PMC10366325; doi:10.1007/s12015-023-10524-2)
Supplement: Supplementary file 2 — Supplementary file2 Supplementary Figure 2: miRNA sequencing reveals novel sets of differentially expressed miRNAs during neural cell fate determination in vitro. (A) qPCR analysis of the expression of pluripotency and differentiation-related miRNAs in hESCs, NSCs, and Diff.NSCs. (B) Complete list of miRNAs specifically differentially expressed in NSCs (Group 1 and Group 2). (C) Complete list of miRNAs upregulated during neural differentiation (Group 3 and Group 4). (D) Complete list of miRNAs downregulated during neuronal differentiation (Group 5 and Group 6). (E) Complete list of miRNAs maintained in stem cells but differentially expressed with terminal differentiation (Group 7 and Group 8). (PDF 542 kb) [file 12015_2023_10524_MOESM2_ESM.pdf]

A

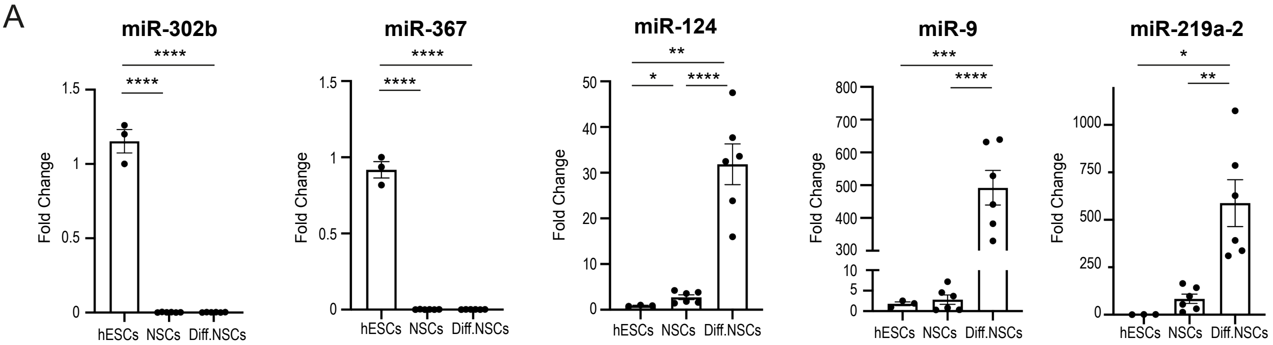

B

miRNAs specifically differentially expressed in NSCs

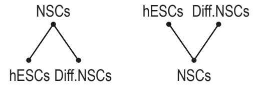

Group 1	Group 2
miR-122-5p	let-7a-3p
miR-125b-1-3p	let-7a-5p
miR-1285-3p	miR-101-3p
miR-130b-5p	miR-1246
miR-132-5p	miR-129-2-3p
miR-181a-2-3p	miR-129-5p
miR-181b-2-3p	miR-1301-3p
miR-1908-5p	miR-135a-2-3p
miR-199b-5p	miR-135a-5p
miR-27b-5p	miR-135b-3p
miR-28-3p	miR-135b-5p
miR-28-5p	miR-139-5p
miR-30c-2-3p	miR-181d-5p
miR-326	miR-182-5p
miR-363-5p	miR-183-5p
miR-424-3p	miR-186-5p
miR-424-5p	miR-19b-3p
miR-450a-5p	miR-210-3p
miR-450b-5p	miR-2113
miR-4705	miR-216a-5p
miR-548ah-5p	miR-3065-3p
miR-548b-5p	miR-3065-5p
miR-550a-3p	miR-32-5p
miR-551b-5p	miR-514a-3p
miR-574-3p	miR-9-3p
miR-760	miR-95-3p
miR-877-5p	
miR-935	

C

miRNAs upregulated during neuronal differentiation

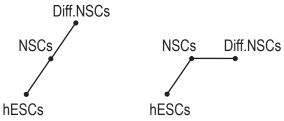

Group 3	Group 4
let-7c-5p	miR-1247-3p
let-7e-5p	miR-1247-5p
miR-100-5p	miR-125b-2-3p
miR-1197	miR-1271-5p
miR-1226-3p	miR-127-3p
miR-125b-5p	miR-130a-5p
miR-127-5p	miR-130b-3p
miR-1299	miR-132-3p
miR-135a-3p	miR-148b-3p
miR-136-3p	miR-149-5p
miR-136-5p	miR-152-3p
miR-137-3p	miR-153-5p
miR-148b-5p	miR-15a-5p
miR-153-3p	miR-181a-3p
miR-154-3p	miR-181b-3p
miR-154-5p	miR-197-3p
miR-16-5p	miR-199a-5p
miR-181a-5p	miR-219a-1-3p
miR-216b-5p	miR-2277-5p
miR-219a-2-3p	miR-23b-3p
miR-219a-5p	miR-27b-3p
miR-299-3p	miR-301a-5p
miR-299-5p	miR-301b-5p
miR-301a-3p	miR-30a-3p
miR-301b-3p	miR-30a-5p
miR-3074-5p	miR-30c-5p
miR-323a-3p	miR-3118
miR-323b-3p	miR-328-3p
miR-329-3p	miR-337-3p
miR-331-3p	miR-339-5p
miR-340-5p	miR-3615
miR-342-3p	miR-363-3p
miR-345-5p	miR-379-3p
miR-3613-5p	miR-409-5p
miR-362-5p	miR-411-3p
miR-369-3p	miR-4286
miR-369-5p	miR-454-5p
miR-370-3p	miR-493-3p
miR-376a-3p	miR-493-5p
miR-376c-3p	miR-500a-5p
miR-377-3p	miR-501-5p
miR-379-5p	miR-505-5p
miR-380-3p	miR-551b-3p
miR-380-5p	miR-651-5p
miR-381-3p	miR-652-5p
miR-382-3p	miR-7706
miR-382-5p	miR-887-3p
miR-409-3p	miR-92b-3p
miR-410-3p	
miR-411-5p	
miR-412-5p	
miR-432-5p	
miR-433-3p	
miR-454-3p	
miR-487a-3p	
miR-487b-3p	
miR-494-3p	
miR-495-3p	
miR-496	
miR-539-3p	
miR-539-5p	
miR-543	
miR-652-3p	
miR-656-3p	
miR-889-3p	

D

miRNAs downregulated during neuronal differentiation

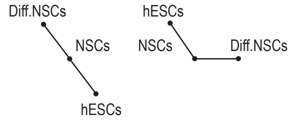

Group 5	Group 6
miR-12135	miR-1264
miR-1275	miR-1283
miR-1303	miR-1298-5p
miR-148a-5p	miR-1323
miR-200b-3p	miR-140-3p
miR-200c-3p	miR-141-3p
miR-296-3p	miR-146a-5p
miR-296-5p	miR-155-5p
miR-302a-3p	miR-17-3p
miR-302a-5p	miR-17-5p
miR-302b-3p	miR-185-5p
miR-302b-5p	miR-187-3p
miR-31-5p	miR-18a-3p
miR-335-3p	miR-18a-5p
miR-367-3p	miR-191-5p
miR-378a-3p	miR-192-5p
miR-425-3p	miR-193b-3p
miR-4454	miR-194-5p
miR-4483	miR-200a-3p
miR-486-5p	miR-200a-5p
miR-653-5p	miR-205-5p
miR-7977	miR-20a-3p
miR-874-3p	miR-20a-5p
miR-92a-1-5p	miR-224-5p
	miR-27a-3p
	miR-302d-5p
	miR-32-3p
	miR-371a-3p
	miR-371a-5p
	miR-372-3p
	miR-372-5p
	miR-373-3p
	miR-374a-3p
	miR-448
	miR-498-5p
	miR-512-3p
	miR-512-5p
	miR-515-5p
	miR-517c-3p
	miR-518b
	miR-518c-3p
	miR-518c-5p
	miR-519d-3p
	miR-520a-3p
	miR-520a-5p
	miR-520d-5p
	miR-520f-3p
	miR-520g-3p
	miR-520g-5p
	miR-522-3p
	miR-525-5p
	miR-526b-5p
	miR-548e-3p
	miR-548f-3p
	miR-590-3p
	miR-629-5p
	miR-653-3p
	miR-767-5p
	miR-96-5p
	miR-9983-3p

E

miRNAs maintained in stem cells but differentially expressed with terminal differentiation.

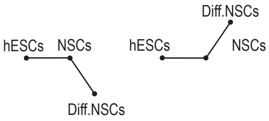

Group 7	Group 8
miR-106b-3p	let-7f-5p
miR-1260b	let-7g-5p
miR-1304-3p	miR-124-3p
miR-151a-5p	miR-130a-3p
miR-16-2-3p	miR-140-5p
miR-204-5p	miR-1843
miR-20b-3p	miR-216a-3p
miR-21-3p	miR-216b-3p
miR-21-5p	miR-26a-5p
miR-222-3p	miR-26b-5p
miR-222-5p	miR-30b-5p
miR-22-3p	miR-330-3p
miR-22-5p	miR-335-5p
miR-25-5p	miR-33a-5p
miR-27a-5p	miR-421
miR-29a-3p	miR-4473
miR-423-3p	miR-499a-5p
miR-4455	miR-500a-3p
miR-455-3p	miR-501-3p
miR-455-5p	miR-532-3p
miR-574-5p	miR-582-3p
miR-6765-3p	miR-582-5p
miR-7974	miR-598-3p
miR-877-3p	miR-625-3p
miR-92a-2-5p	miR-628-5p
miR-92b-5p	miR-660-5p
miR-941	miR-7-1-3p
	miR-9-5p

Supplementary Figure 2
