# Supplementary material for: MicroRNA Profiling of Self-Renewing Human Neural Stem Cells Reveals Novel Sets of Differentially Expressed microRNAs During Neural Differentiation In Vitro
Source: Stem Cell Rev Rep. 2023 Mar 14;19(5):1524–39. doi: 10.1007/s12015-023-10524-2 (PMC10366325; doi:10.1007/s12015-023-10524-2)
Supplement: Supplementary file 3 — Supplementary file3 Supplementary Figure 3: miRNA clusters enriched in NSCs share the seed sequence with cell cycle regulatory miRNAs in pluripotent hESCs and are directly transcriptionally regulated by c-MYC (A) Expression of selected clusters enriched in each cell type throughout the neural differentiation in hESCs, NSCs, and Diff.NSCs. (B) Table of miRNAs included in each cluster. (C) Representation of miRNA families in hESCs, NSCs, and Diff.NSCs (PDF 1743 kb) [file 12015_2023_10524_MOESM3_ESM.pdf]

A

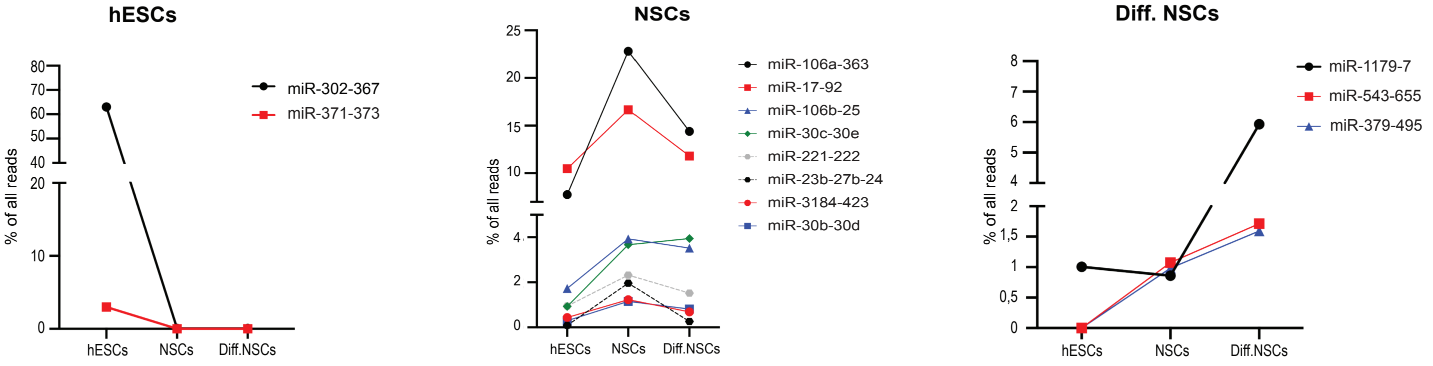

B

cluster	miRNAs in cluster
miR-106a-363	miR-106a-3p, miR-106a-5p, miR-18b-3p, miR-18b-5p, miR-19b-2-5p, miR-19b-3p, miR-20b-3p, miR-20b-5p, miR-363-3p, miR-363-5p, miR-92a-2-5p, miR-92a-3p
miR-106b-25	miR-106b-3p, miR-106b-5p, miR-25-3p, miR-25-5p, miR-93-3p, miR-93-5p
miR-1179-7	miR-1179, miR-3529-5p, miR-7-2-3p, miR-7-5p
miR-17-92	miR-17-3p, miR-17-5p, miR-18a-3p, miR-18a-5p, miR-19a-3p, miR-19a-5p, miR-19b-1-5p, miR-19b-3p, miR-20a-3p, miR-20a-5p, miR-92a-1-5p, miR-92a-3p
miR-221-222	miR-221-3p, miR-221-5p, miR-222-3p, miR-222-5p
miR-23b-24b-27	miR-23b-3p, miR-23b-5p, miR-24-1-5p, miR-24-3p, miR-27b-3p, miR-27b-5p, miR-3074-5p
miR-302-367	miR-302a-3p, miR-302a-5p, miR-302b-3p, miR-302b-5p, miR-302d-5p, miR-367-3p, miR-367-5p
miR-30b-30d	miR-30b-3p, miR-30b-5p, miR-30d-3p, miR-30d-5p
miR-30c-30e	miR-30c-1-3p, miR-30c-5p, miR-30e-3p, miR-30e-5p
miR-3184-423	miR-3184-5p, miR-423-3p, miR-423-5p
miR-371-373	miR-371a-3p, miR-371a-5p, miR-372-3p, miR-372-5p, miR-373-3p, miR-373-5p
miR-379-495	miR-1197, miR-299-3p, miR-299-5p, miR-300, miR-323a-3p, miR-323a-5p, miR-329-3p, miR-329-5p, miR-376a-3p, miR-376a-5p, miR-376a-2-5p, miR-376b-3p, miR-376c-3p, miR-379-3p, miR-379-5p, miR-380-3p, miR-380-5p, miR-411-3p, miR-411-5p, miR-494-3p, miR-494-5p, miR-495-3p, miR-495-5p, miR-543, miR-654-3p, miR-654-5p, miR-758-5p
miR-543-655	miR-1185-1-3p, miR-1185-5p, miR-1185-2-3p, miR-300, miR-376a-3p, miR-376a-5p, miR-376a-2-5p, miR-376b-3p, miR-376c-3p, miR-381-3p, miR-381-5p, miR-487b-3p, miR-487b-5p, miR-494-3p, miR-494-5p, miR-495-3p, miR-495-5p, miR-539-3p, miR-539-5p, miR-543, miR-544a, miR-654-3p, miR-654-5p, miR-655-3p, miR-655-5p, miR-889-3p, miR-889-5p

C

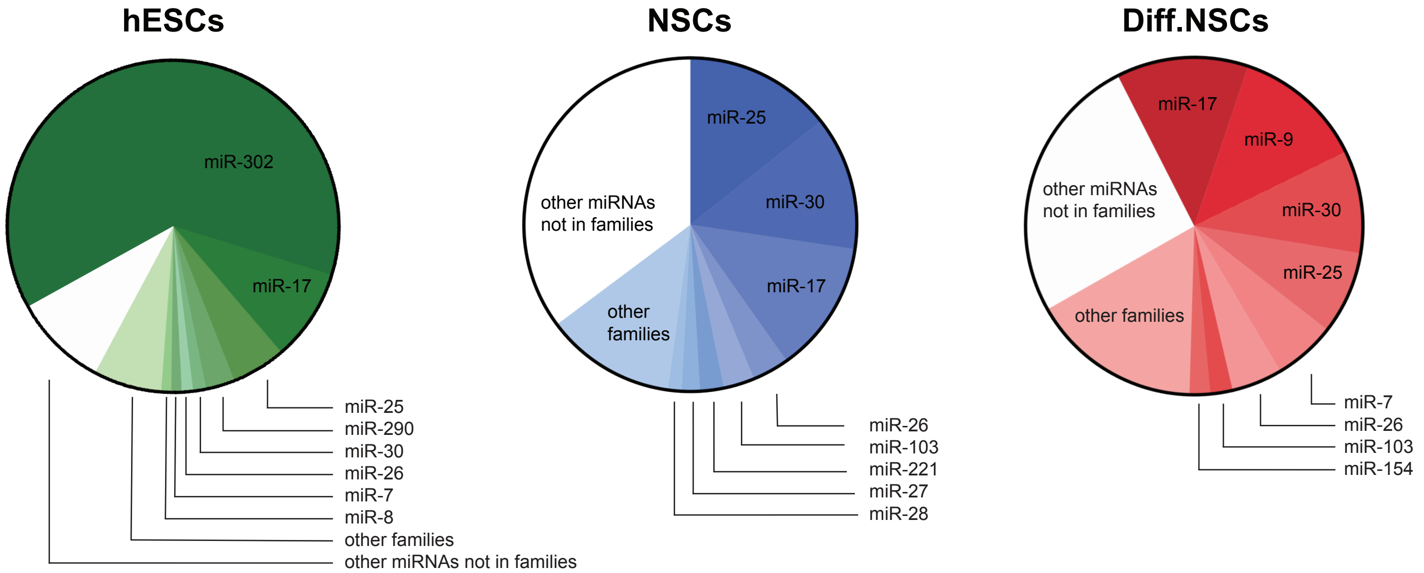

Supplementary Figure 3
