# Supplementary material for: MicroRNA Profiling of Self-Renewing Human Neural Stem Cells Reveals Novel Sets of Differentially Expressed microRNAs During Neural Differentiation In Vitro
Source: Stem Cell Rev Rep. 2023 Mar 14;19(5):1524–39. doi: 10.1007/s12015-023-10524-2 (PMC10366325; doi:10.1007/s12015-023-10524-2)
Supplement: Supplementary file 4 — Supplementary file4 (DOCX 6956 kb) [file 12015_2023_10524_MOESM4_ESM.docx]

**Supplementary material:**

Supplementary Table 1: List of primers and probes

| **Protein-coding GENE** | **NCBI ID** | **FORWARD SEQUENCE** | **REVERSE SEQUENCE** |
| --- | --- | --- | --- |
| *DCX* | 1641 | TATGCGCCGAAGCAAGTCTCCA | CATCCAAGGACAGAGGCAGGTA |
| *GAPDH* | 2597 | AGCCACATCGCTCAGACAC | GCCCAATACGACCAAATCC |
| *MAP2* | 4133 | TTGGTGCCGAGTGAGAAGA | GTCTGGCAGTGGTTGGTTAA |
| *NANOG* | 79923 | TTTGTGGGCCTGAAGAAAACT | AGGGCTGTCCTGAATAAGCAG |
| *POU5F1* | 5460 | CTGGGTTGATCCTCGGACCT | CCATCGGAGTTGCTCTCCA |
| *SOX1* | 6656 | TCCCCCGCGTGAACTG | CAAGGCATTTTGCGTTCACA |
| *SOX2* | 6657 | TACAGCATGTCCTACTCGCAG | GAGGAAGAGGTAACCACAGGG |
| *TUBB3* | 10381 | TCAGCGTCTACTACAACGAGGC | GCCTGAAGAGATGTCCAAAGGC |
| *Apex1* |  | GTGTCACACAATGTGCTGTGC | AACCCCGTATCTGCACCG |
| *E-Box 1 in miR17-92 promoter region* |  | ACCTCGGAAACCCACCAAG | TCTCCCTGGGACTCGACG |
| *E-Box 2 in miR17-92 promoter region* |  | CCAAGCTGAAGTACAGGCAAACT | TGGGTGGTCTAACCTAGTGTTATGG |
| *E-Box 3 in miR17-92 promoter region* |  | TTGTCACTACAGATGGTCTAAAGGTTACTT | TCCTTGTCTCCACTTCCCCA |
| *E-Box 4 in miR17-92 promoter region* |  | TTTAAACAGGATATTTACGTTCTGC | GAGGAAATCTTCACATCCACG |
|  | | | |
| **miRNA** | **Source** | | **IDENTIFIER (Probe ID)** |
| *hsa-miR-9-5p* | *Thermo Fisher Scientific* | | *000583* |
| *hsa-miR-124* | *Thermo Fisher Scientific* | | *001182* |
| *hsa-miR-145* | *Thermo Fisher Scientific* | | *002278* |
| *hsa-miR-302b* | *Thermo Fisher Scientific* | | *00531* |
| *hsa-miR-367* | *Thermo Fisher Scientific* | | *555* |

Supplementary Table 2: List of antibodies used for Western blotting and Immunocytochemistry

| **Antibodies (WB)** | **SOURCE** | **IDENTIFIER** |
| --- | --- | --- |
| β-Actin (8H10D10) | Cell Signaling Technology | Cat# 3700 |
| c-MYC | Cell Signaling Technology | Cat# 5605 |
| Cyclin D1 | Cell Signaling Technology | Cat# 55506 |
| Cyclin E1 (HE12) | Santa Cruz Biotechnologz | sc-247 |
| p27 (F-8) | Santa Cruz Biotechnologz | sc-1641 |
| Rb (IF8) | Santa Cruz Biotechnologz | sc-102 |
| pRb (E10) | Santa Cruz Biotechnologz | sc-271930 |
|  | | |
| **Antibodies (ICC)** | **SOURCE** | **IDENTIFIER** |
| NESTIN | Mercs Millipore | MAB5326 |
| MAP2 (D5G1) | Cell Signaling Technology | Cat# 8707 |
| SOX2 (D6D9) | Cell Signaling Technology | Cat# 3579 |
| TUJ (TU-20) | Cell Signaling Technology | Cat# 4466 |

Supplementary table 4: Criteria for individual miRNA categorization

| **Category** | **Group** | **Trend** | **p-adj** | **Fold Change** |
| --- | --- | --- | --- | --- |
| miRNAs differentially expressed specifically in self-renewing NSCs | 1 |  | p-adj1<0.05  p-adj2<0.05 | log2FoldChange1> +0.6  log2FoldChange2< -0.6 |
|  | 2 |  | p-adj1<0.05  p-adj2<0.05 | log2FoldChange1< -0.6  log2FoldChange2> +0.6 |
| miRNAs differentially expressed during neural differentiation | 3 |  | p-adj1<0.05  p-adj2<0.05 | log2FoldChange1> +0.6  log2FoldChange2> +0.6 |
|  | 4 |  | p-adj1<0.05  p-adj2>0.05  p-adj1<0.05  p-adj2<0.05 | log2FoldChange1> +0.6  log2FoldChange2> +0.6  log2FoldChange1> +0.6  log2FoldChange2< +0.6 |
|  | 5 |  | p-adj1<0.05  p-adj2<0.05 | log2FoldChange1< -0.6  log2FoldChange2< -0.6 |
|  | 6 |  | p-adj1<0.05  p-adj2>0.05  p-adj1<0.05  p-adj2<0.05 | log2FoldChange1< -0.6  log2FoldChange2< -0.6  log2FoldChange1< -0.6  log2FoldChange2> -0.6 |
| miRNAs maintained in stem cells but differentially expressed with the onset of terminal differentiation | 7 |  | p-adj1>0.05  p-adj2<0.05  p-adj1<0.05  p-adj2<0.05  p-adj1>0.05  p-adj2<0.05 | log2FoldChange1< +0.6  log2FoldChange2< -0.6  log2FoldChange1> -0.6∧< +0.6  log2FoldChange2< -0.6  log2FoldChange1> +0.6  log2FoldChange2< -0.6 |
|  | 8 |  | p-adj1<0.05  p-adj2<0.05  p-adj1>0.05  p-adj2<0.05  p-adj1>0.05  p-adj2<0.05 | log2FoldChange1> -0.6∧< +0.6  log2FoldChange2> +0.6  log2FoldChange1> +0.6  log2FoldChange2> +0.6  log2FoldChange1< +0.6  log2FoldChange2> +0.6 |
